# The Role of the *si*-Face Tyrosine of a Homodimeric Ferredoxin-NADP^+^ Oxidoreductase from *Bacillus subtilis* during Complex Formation and Redox Equivalent Transfer with NADP^+^/H and Ferredoxin

**DOI:** 10.3390/antiox12091741

**Published:** 2023-09-08

**Authors:** Daisuke Seo

**Affiliations:** Division of Material Science, Graduate School of Natural Science and Technology, Kanazawa University, Kakuma, Kanazawa 920-1192, Japan; dseo@se.kanazawa-u.ac.jp; Tel.: +81-76-264-5683

**Keywords:** ferredoxin, charge transfer complex, FNR, stopped-flow spectrophotometry

## Abstract

In the crystal structure of ferredoxin-NADP^+^ oxidoreductase from *Bacillus subtilis* (*Bs*FNR), Tyr50 stacks on the *si*-face of the isoalloxazine ring portion of the FAD prosthetic group. This configuration is highly conserved among the homodimeric ferredoxin-NAD(P)^+^ oxidoreductases (FNR) from Gram-positive bacteria and photosynthetic bacteria. In this report, pre-steady state reactions of Tyr50 variants with NADP^+^/NADPH and ferredoxin from *B. subtilis* (*Bs*Fd) were examined with stopped-flow spectrophotometry to assess the effects of the mutation on the formation of FNR-substrate complexes and following redox equivalent transfer. Mixing oxidized *Bs*FNRs with NADPH resulted in a rapid complex formation followed by a rate-limiting hydride transfer. The substitution substantially modulated the intensity of the charge transfer absorption band and decreased the observed hydride transfer rates compared to the wild type. Reduction of the Y50W mutant by NADPH proceeded in a monophasic manner, while the Y50G and Y50S mutants did in biphasic phases. The reduced Tyr50 mutants hardly promoted the reduction of NADP^+^. Mixing oxidized *Bs*FNRs with reduced *Bs*Fd resulted in the reduction of the FNRs. The observed FNR reduction rates of the three variants were comparable, but in the Y50G and Y50S mutants, the amount of the reduced FNR at the rapid phase was decreased, and a slow FNR reduction phase was observed. The obtained results suggest that the replacements of Tyr50 with Gly and Ser permitted the conformational change in the reduced form, which induced an asymmetric kinetic behavior between the protomers of the homodimeric *Bs*FNR.

## 1. Introduction

Ferredoxin (Fd) is a small iron sulfur protein functioning as a redox mediator in various metabolic processes such as photosynthetic NAD(P)^+^ reduction, assimilations of nitrogen and sulfur, cyclic electron transfer in chloroplast, anaerobic carbon fixation, cytochrome P450-dependent hydroxylation, radical *s*-adenosylmethionine (SAM)-dependent reactions [1,2,3,4,5,6,7,8]. Fd also participates in iron homeostasis and maturation of iron-sulfur proteins [1,9]. Because the Fd-dependent processes are involved in the synthesis and repair of the fundamental cellular materials, such as DNA, heme, lipid and iron sulfur cluster, as well as donation of redox equivalents to ROS scavengers, Fd and its partner enzymes play a crucial role in the tolerance to oxidative stresses [4,9,10]. In many living organisms, ferredoxin-NAD(P)^+^ oxidoreductase ([EC 1.18.1.2][EC 1.18.1.3], FNR) catalyzes the reduction/oxidation of Fd using NAD(P)^+^/H.

NAD(P)H + 2Fd_ox_ ⇆ NAD(P)^+^ + 2Fd_red_ + H^+^.

**Figure 1:**
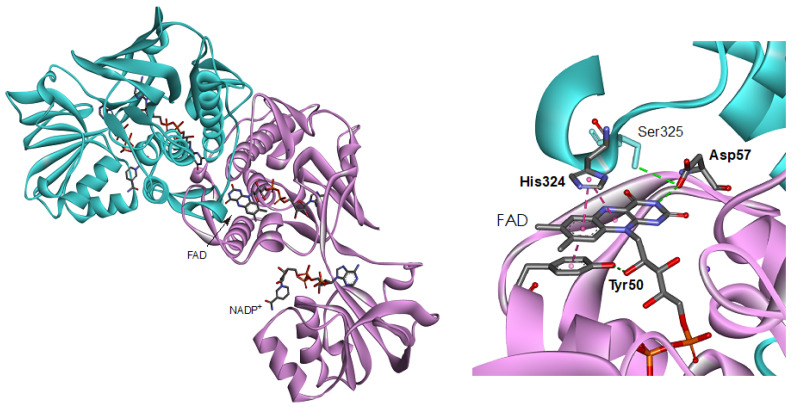
Overall structure (**left**) and close-up view around the isoalloxazine ring moiety (**right**) of the *Bs*FNR homodimer (PDB ID: 3LZX). Protomer A and B are colored with pink and blue, respectively.

FNR is a member of the electron transfer dehydrogenase of flavoprotein superfamily, which contains a flavin adenine dinucleotide (FAD), or a flavin mono nucleotide (FMN) as the prosthetic group [2,3,11,12,13]. The crystal structures of FNRs from firmicutes *Bacilli* [14,15], alpha-proteobacterium *Rhodopseudomonas palustris* [16] and thermophilic bacterium *Thermus thermophiles* ([17], PDB ID: 2ZBW) have revealed a conservation of their structural topology with the bacterial NADPH-thioredoxin reductase (TrxR) [18], herein referred to as a TrxR-type FNR. TrxR-type FNRs possess a distinctive structural fold from the other types of FNR and its isoforms such as FNRs from plant and cyanobacteria, adrenodoxin reductase and putidaredoxin reductase [2,3,6,11,12,13,19]. Despite the difference in the protein fold, a similar arrangement of the amino acid residues is frequently found around the isoalloxazine ring portion in flavoproteins, including FNRs. In plant-type FNR, its superfamily enzymes and flavodoxins aromatic residues stacked on the *re*- and *si*-faces of the isoalloxazine ring portion have been reported to be crucial to their functions. For example, in methionine synthase reductase and cytochrome P450 reductase the *re*-face Trp residues are involved in the controls of the accessibility of the nicotine amide ring portion of NADP^+^/H to the isoalloxazine ring portion and inter flavin electron transfer [20]. In the recent AFM work on *Anabaena* FNR, the bipartite binding/dissociation mode of NADP^+^ has been confirmed where the C-terminus Tyr regulate the access, release and orientation of the nicotinamide ring moiety to the isoalloxazine ring [21]. In the case of TrxR-type FNRs, replacements of the *re*-face His and Tyr residues modulate the turnover rates and affinity toward NADPH [14,16]. On the *si*-face of the isoalloxazine ring portion, tyrosine residue is stacked [22,23,24,25,26,27,28]. The replacements of the *si*-face Tyr residues in plant-type FNRs decrease thermal stability, affinity toward NADP^+^/H and turnover rate of the reaction with NADPH [22,23]. In the case of cytochrome *b*_5_ reductase [24,25] and flavodoxins [26,27,28], the replacements affect the redox potentials of the FAD and FMN prosthetic groups. In *Anabaena* flavodoxin, the presence of Tyr on the *si*-face increases the stability of FMN binding [28]. In the crystal structure of *Bacillus subtilis* FNR (*Bs*FNR), Tyr50 stacks on the *si*-face of the ring almost in parallel at a distance 3.3 Å and hydrogen bonds with 2′-OH of the ribityl moiety of FAD prosthetic group (Figure 1) [14]. In the previous steady-state reaction analyses of Tyr50 mutants of *Bs*FNR, the turnover rates of the diaphorase assay using NADPH and ferricyanide, and a cytochrome *c* reduction assay using *Bs*Fd as an electron acceptor were drastically decreased, especially by the replacements with the non-aromatic residues [29].

During the reaction catalyzed by FNR, a hydride transfer reaction with NAD(P)^+^/H and electron transfer reaction with Fd occur via the formation of the competent FNR-substrate complexes [30,31,32,33,34]. In the complexes, precise arrangements of the reacting atoms/groups of the cofactors and amino acid residues are indispensable to optimize the redox properties of the cofactors and tunneling of hydride and electron [30,31,32,33,34,35]. In *Bs*FNR the *si*-face Tyr50 may not contact directly with the substrate molecule NADP^+^/H in the competent substrate-FNR complex, but previous steady-state measurements indicated that its replacements modulated the spectroscopic and catalytic properties toward formation of the complex with NADP^+^/H [29]. Molecular dynamics simulations of wild type (WT) *Bs*FNR and its Y50W mutant suggest that the replacement induces a drastic motion of the isoalloxazine ring portion and, thus, may fracture the interaction of amino acid residues and isoalloxazine ring with substrate molecules [36]. Our previous stopped-flow studies with WT *Bs*FNR revealed that, in the reduction with NADPH, rapid charge transfer (CT) complex formation followed by a rate-limiting hydride transfer occurs, and in the reduction with reduced *Bs*Fd, rapid formation of semiquinone followed by a slower transition to the hydroquinone take place [37]. In this report, details of the effects of the replacement of Tyr50 on the formation of FNR substrate complexes and the following redox equivalent transfer are explored with the same kinetic approach. The replacement of Tyr50 with Trp retained similar kinetic behavior as WT *Bs*FNR, though the observed hydride transfer rate was decreased significantly, whereas those with Gly and Ser exhibited a biphasic kinetic behavior which may related to the asymmetry in the conformation of protomers induced by the reduction of FAD.

## 2. Materials and Methods

### 2.1. Preparations of Tyr50 BsFNR Mutants and BsFd

Expression and purification of the Tyr50 mutants, Y50G, Y50S and Y50W *Bs*FNRs, were performed, according to the method described in [29]. Briefly, a mutated *Bs*FNR gene in pETBlue I vector was expressed in *E. coli* Tuner(DE3)pLacI cells (Novagen, Merck KGaA, Darmstadt, Germany). Mutated *Bs*FNR was purified from the cell extract by ammonium sulfate fractionation, DyeMatrex Red A affinity gel, hydroxyapatite and anion exchange column chromatography. The purified *Bs*FNR was dialyzed against 20 mM 4-(2-hydroxyethyl)-1-piperazineethanesulfonic acid (HEPES)-NaOH buffer (pH 7.0) and stored at −80 °C before use.

WT *B. subtilis* Fd (*Bs*Fd) was prepared basically according to the method in [38]. Briefly, *Bs*Fd gene in pHY300PLK vector was expressed in *E. coli* Novablue cells (Novagen). *Bs*Fd in the cell extract was purified by ammonium sulfate fractionation, first anion exchange using DEAE Sephadex A-25 gel in place of DEAE Sepharose CL-6B gel (Cytiva, Global Life Sciences Technologies, Japan K.K., Tokyo, Japan), hydrophobic interaction, the second anion exchange and gel permeation column chromatography. Purified *Bs*Fd solution was stored at −80 °C before use.

### 2.2. Stopped-Flow Spectrophotometry

Pre-steady-state reaction was monitored using a stopped-flow spectrophotometer system equipped with a 150 W xenon discharge lump and a photodiode array detector (Unisok Ltd., Osaka, Japan) [37]. To decrease the photoexcitation of the Y50 mutants, the measuring light was provided after passing through a long pass (L-39, Toshiba, Japan) and FL-W (Kenko-Tokina, Tokyo, Japan) optical filters. The measurements were performed under anaerobic conditions (<20 ppm O_2_) in a glove box (UNICO LTD., Tsukuba, Japan) with a nitrogen atmosphere containing hydrogen gas (<5%) to remove residual dioxygen in combination with a palladium catalyst [37]. The buffers, protein solutions and reagents were treated with more than 5 cycles of evacuation and purge with pure argon gas. All the buffer, protein and substrate concentrations are indicated as final concentrations after mixing unless otherwise noted. All the measurements were performed in 20 mM HEPES-NaOH buffer (pH 7.0) at 10 °C. The average of five replicates were utilized for the kinetic analyses unless otherwise noted. To prepare the samples, purified deionized water was used (Simplicity UV, Milli-Q, Merck KGaA). HEPES, sodium hydroxide (GR grade) and ethylenediaminetetraacetic acid disodium salt dihydrate (EDTA 2Na, nuclease and protease tested) were purchased from Nacalai tesque (Kyoto, Japan). NADP^+^ and NADPH were purchased from Oriental yeast Co., Ltd. (Tokyo, Japan).

The reduced *Bs*FNR was prepared as follow: sodium dithionite and methyl viologen were added to the approximately 200 μM *Bs*FNR in 20 mM HEPES-NaOH buffer (pH 7.0) at the final concentrations of 4.0 mg/mL and 10 µM, respectively. After incubation for several minutes at 5 °C, the solvent was exchanged by a size exclusion chromatography (Sephadex G-25, Cytiva) for 20 mM HEPES-NaOH buffer (pH 7.0) containing 0.32 μg/mL sodium dithionite. All the process was performed under anaerobic conditions in the glove box. Concentration of the reduced FNR was estimated in oxidized form after reoxidation by air exposition.

The reduced *Bs*Fd was prepared using sodium dithionite as reductant under anaerobic conditions in the glove box. Briefly, sodium dithionite was added to approximately 1 mM *Bs*Fd solution at the final concentration of 4.0 mg/mL. After standing for several minutes at 5 °C the solvent was exchanged for 20 mM HEPES NaOH buffer (pH 7.0) containing 0.32 μg/mL sodium dithionite using size exclusion chromatography (Sephadex G-25). The concentration of the reduced *Bs*Fd was estimated in oxidized form after reoxidation by air exposition.

(4*S*-^2^H)-NADPD (*S*-NADPD) was prepared as described in [37].

Global analysis of the transient spectra was performed using two ([A]→[B]) or three ([A]→[B]→[C] (fast/slow)) components’ sequential reaction model in the Olis GlobalWorks software (ver. 5.888 309, OLIS Inc., Athens, GA, USA). In this manuscript, rate constant (*k*) and amplitude (*A*) of each kinetic phase are designated as *k*_I_ and *A*_I_ for phase I ([A]→[B]), and *k*_II_ and *A*_II_ for phase II ([B]→[C]) in both models. The transient spectral data of 400–800 nm wavelength region were utilized for the analyses. For the analyses of the reaction with NADPH, time region of 0–1000 ms for Y50G mutant and that of 0–2000 ms for Y50S and Y50W mutants were utilized. For the analyses of FNR reduction by reduced Fd, time region of 0–100 ms was utilized. The kinetic analyses of the absorbance at 460 nm were performed in Igor Pro software (Versions 6.3 and 9.0, Wavemetrics Inc., Portland, OR, USA) using single- or double-exponential decay function.

### 2.3. Spectroscopic Measurements and Protein Structure Representation

The UV-visible absorption spectra were measured with a double beam spectrophotometer (V-560, JASCO, Tokyo, Japan) at room temperature (20–25 °C). Protein subunit and substrate concentrations were determined using the extinction coefficients for Y50G and Y50S *Bs*FNRs (ε_457_ = 12.7 mM^−1^ cm^−1^ [29]), Y50W *Bs*FNR (ε_458_ = 11.8 mM^−1^ cm^−1^ [29]), *Bs*Fd (ε_390_ = 16.0 mM^−1^ cm^−1^ [39]), and NADPH (ε_340_ = 6.2 mM^−1^ cm^−1^). NADP^+^ concentration was determined in reduced NADPH form after incubation for several minutes in the presence of 10 mM glucose-6-phosphate (Oriental yeast Co., Ltd.) and 5 U/mL glucose-6-phasphate dehydrogenase (from *Leuconostoc mesenteroides*, Worthington Biochemicals Co., Ltd., Lakewood, NJ, USA).

The representation of the protein 3D-structure was performed with BIOVIA Discovery Studio Visualizer (Ver. 21.1, Dassault Systèms, Vélizy-Villacoublay, France).

## 3. Results

### 3.1. Reduction of Oxidized BsFNRs with NADPH

**Figure 2:**
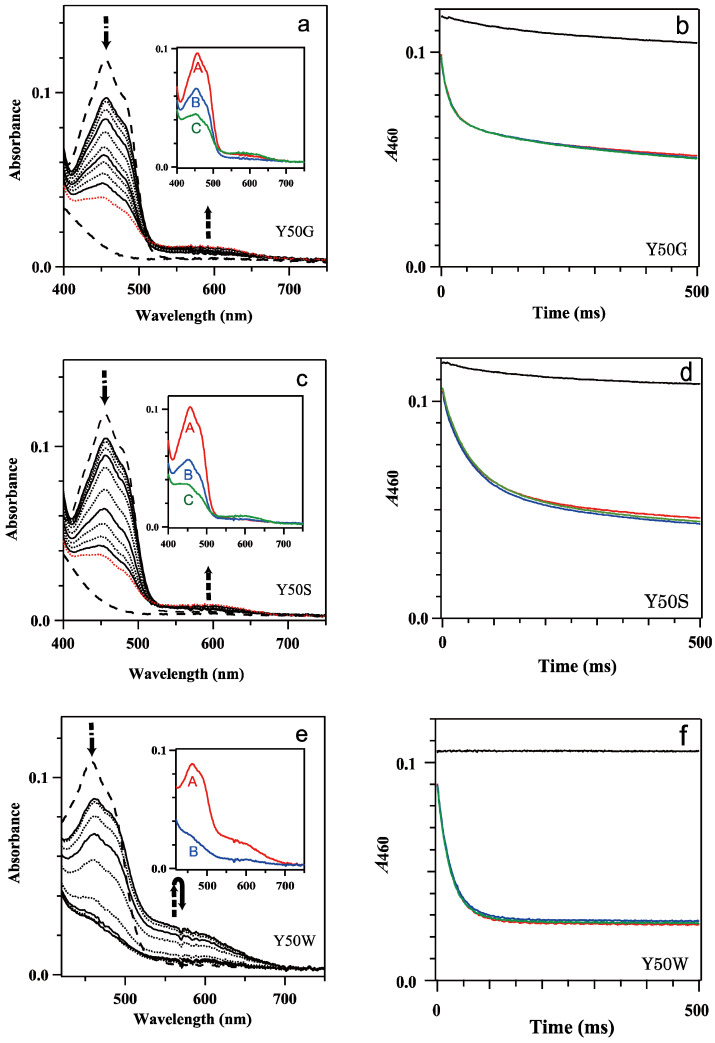
(**a**,**c**,**e**) Transient absorption spectra of the reaction of mixing oxidized 9.1 μM Y50G (**a**), 9.1 μM Y50S (**c**) and 8.9 μM Y50W (**e**) *Bs*FNR mutants with 100 μM NADPH in 20 mM HEPES-NaOH buffer at 10 °C. Thick broken lines indicate the spectra of the oxidized and fully reduced states. Thin continuous lines represent the spectra at 1, 10, 100 and 1000 ms, and thin dotted lines at 2, 5, 20, 50, 200, 500 and 2000 ms from top to bottom at 450 nm. In (**a**,**c**) the spectra at 2000 ms are indicated with red dotted lines. The insets of (**a**,**c**,**e**) show the spectrum of each kinetic component estimated by a global analysis of the transient spectra with a one or two-step sequential reaction model (([A]→[B]) or ([A]→[B]→[C] (fast/slow))). (**b**,**d**,**f**) Time-dependent absorbance changes at 460 nm of mixing Y50G (**b**), Y50S (**d**) and Y50W (**f**) mutants with 0 (black), 100 (red), 200 (blue) and 500 μM (green) NADPH. The data are an average of five replicates.

The reaction of oxidized 9.1 μM Y50G, 9.1 μM Y50S and 8.9 μM Y50W *Bs*FNR mutants with 0, 100, 200, 300 and 500 μM NADPH was monitored under anaerobic conditions (Figure 2). Mixing Y50G and Y50S mutants with 0 μM NADPH (i.e., mixing FNR solution with 20 mM HEPES-NaOH buffer) exhibited absorption change with time (Figure 2b,d and Figure S1a,b), whereas the Y50W mutant provided almost no change during the period monitored (0–4 s) (Figure 2f and Figure S1c). An appearance of the broad absorption band with a maximum at around 600 nm corresponds to the formation of a neutral semiquinone (Figure S1a,b). The formation of the semiquinone state under the experimental conditions is due to the photoreduction of the Y50G and Y50S mutants which lack the aromatic residue on the *si*-face, Tyr50 in WT or Trp50 in Y50W mutant, under the irradiation of white light as the measuring light in the stopped-flow spectrophotometer. These aromatic residues function as an electron donor of the photoexcited FAD, then a rapid recombination between FAD anion radical and Tyr/Trp cation radical occurred [36]. It has been reported that morpholine-based buffers and HEPES act as a sacrificial electron donor of the light-excited flavins [40,41]. Under the experimental conditions HEPES will act as the sacrificial electron donor, which proceeds an accumulation of the photoreduced Y50G and Y50S *Bs*FNRs species. To confirm a photo-accumulation of the semiquinone form and estimate a spectrum of the semiquinone state of Y50G mutant, a measurement was performed in the presence of EDTA (Figure S2). Obtained transient spectra indicated that the transition of oxidized Y50G to semiquinone state occurred rapidly as an increase in the intensity of the absorption band with a maximum at around 600 nm was observed. The photoreduction proceeded further to a formation of the fully reduced hydroquinone state (Figure S2). In this work, to diminish the photoreduction, measuring light was passed through a long pass optical filter as described in Section 2 Materials and Methods.

Mixing Y50G, Y50S and Y50W *Bs*FNRs_ox_ with 100 to 500 μM NADPH exhibited a drop of the absorbance of the flavin absorption band I with a peak at around 460 nm in the spectra at 1 ms after mixing (Figure 2a,c,e). A gradual decrease of the absorption intensity was followed with time. In Y50W mutant the absorbance became almost stable at 200 ms after mixing (Figure 2e,f). In contrast, the decrease of the absorbance of flavin band I of Y50G and Y50S mutants continued beyond 1 s (Figure 2a–d).

In the charge transfer (CT) absorption band region (500–700 nm) [37], an increase of the absorbance was observed in the spectra at 1 ms for all the mutants, but their intensities depended on the residue replaced (Figure 2a,c,e). Y50W mutant provided a maximum intensity at 1 ms, then the absorption decreased with time to close to the intensity before mixing at 100 ms (Figure 2e). In contrast, the intensities of Y50G and Y50S mutants at 1 ms were very low (Figure 2a,c). At 1000 ms after mixing, the absorbance slightly increased, which would be due to the formation of semiquinone by the photoreduction as described in the previous paragraph rather than the formation of CT complex. When *S*-NADPD was utilized in place of NADPH, the maximum intensities of the CT bands of Y50G and Y50W mutants were comparable to those with NADPH, while the observed rate for the reduction of FAD prosthetic group decreased significantly (Figure 2a,e and Figure S3a,b, Table 1). This result indicates that CT formation was rapid compared to the following hydride transfer, because the rate of the hydride transfer did not affect significantly on the absorption intensity of the CT band [37].

Global analysis of the time-resolved absorption spectra implied that the spectral transition was approximated by a one-step reaction model ([A]→[B]) for Y50W mutant (inset of Figure 2e) and a two-step sequential reaction model ([A]→[B]→[C] (fast/slow)) for Y50G and Y50S mutants (inset of Figure 2a,c). Absorption changes at 460 nm were also approximated with the same kinetic models and similar rate constant values (Table 1, Figure S4). It should be noted that the *k*_II_ and *A*_II_ values of Y50G and Y50S mutants (Table 1) contain significant error because the absorbance changes continued beyond the period monitored.

In Y50W mutant (Figure 2e) FNR was almost fully reduced at the equilibrium just as in the case of WT *Bs*FNR [37]. The observed rate constant and amplitude of phase I (*k*_I_ and *A*_I_, respectively) were almost independent from the NADPH concentrations used (100–500 μM) (Table 1).

In the case of Y50G and Y50S mutants, the values for the kinetic constants of both phases (*k*_I_, *A*_I_, *k*_II_ and *A*_II_) were almost independent from NADPH concentration (Table 1). Because the absorbance at around 600 nm was lower compared to those in the absence of NADPH (Figure 2a,c and Figure S1a,b), most part of the transition corresponded to the FNR reduction by NADPH. Considering that the reaction did not reach to the equilibria during the period monitored, actual *A*_II_ values of both mutants would be larger and comparable to the *A*_I_ values (Figure 2a,c and Table 1). The spectra of each kinetic components obtained by the global analysis indicated that both phases represented the reduction of oxidized FNR to fully reduced hydroquinone form (insets of Figure 2a,c).

### 3.2. Oxidation of Reduced BsFNRs with NADP^+^

In 20 mM HEPES-NaOH buffer at pH 7.0 and 10 °C, reduced Y50G, Y50S and Y50W mutants provided absorption spectra with no apparent peak in the 400–600 nm region (Figure S5) in contrast with the result of the WT *Bs*FNR, where an absorption band with a maximum at around 450 nm was observed [37,42].

Mixing reduced 7.1 μM Y50G and 9.1 μM Y50S mutants with 100 and 500 μM NADP^+^ provided almost no absorption change in the transient spectra from 0 to 1 s (Figure S5a,b). When mixing reduced 6.9 μM Y50W mutant with 100 and 500 μM NADP^+^, a slight increase was observed after mixing (Figure S5c), then the absorbance in the flavin absorption band I region increased very slowly and continually with time (inset of Figure S5c). The difference spectrum subtracting the spectrum at 1 ms from at 2 s indicated that this phase corresponded to the oxidation of the FAD prosthetic group (red line in Figure S5c).

### 3.3. Reduction of Oxidized BsFNR Mutants with Reduced BsFd

**Figure 3:**
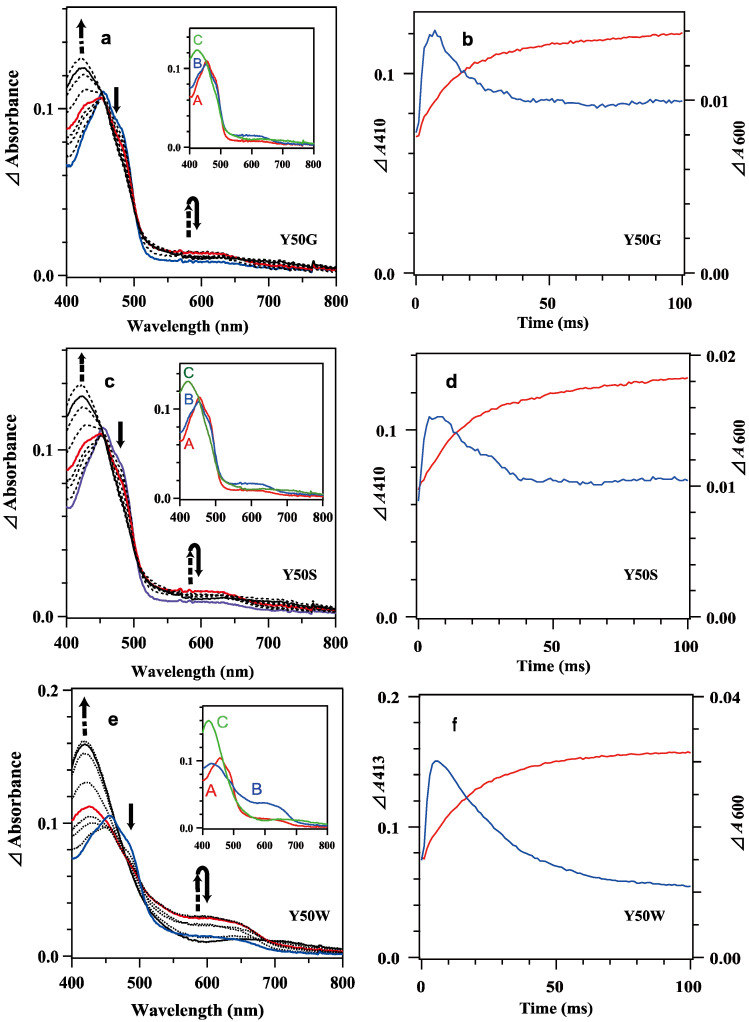
Transient difference absorption spectra in the reaction of mixing oxidized 7.8 μM Y50G (**a**), 8.4 μM Y50S (**c**) and 8.9 μM Y50W (**e**) *Bs*FNR mutants with reduced 74 μM *Bs*Fd in 20 mM HEPES-NaOH buffer (pH 7.0) containing 0.16 μg/mL sodium dithionite at 10 °C. Continuous lines correspond to the difference spectra at 1, 10 and 100 ms and dotted lines at 2, 5, 7, 20, 50 and 200 ms from bottom to top at 410 nm. The spectra at 1 ms and 10 ms are indicated in blue and red, respectively. The insets show the spectra of kinetic components resulting from a global analysis of the transient difference spectra with a two-step sequential reaction model ([A]→[B]→[C] (fast/slow)). (**b**,**d**,**f**) Time dependency of the *Δ*absorbances at 410 or 413 nm (red lines) and 600 nm (blue lines) of Y50G (**b**), Y50S (**d**) and Y50W (**f**) mutants. Details of the calculation of the difference spectra were described in the main text. The data are an average of five replicates.

The reduction of oxidized *Bs*FNR mutants with excess reduced *Bs*Fd was monitored under similar conditions reported for the measurement of WT *Bs*FNR [37]. Because reduced *Bs*Fd exhibited broad absorption in UV–visible region (Figure S6), the absorption spectrum of the reduced *Bs*Fd was subtracted from each transient spectrum to magnify the spectral changes corresponding to the redox reaction between *Bs*FNR and *Bs*Fd (Figure 3a,c,e). The obtained difference spectra contained the spectral changes for the transitions of the redox states of *Bs*Fd as well as *Bs*FNR; thus, difference spectra of ([*Bs*FNR_sq_] + [*Bs*FNR_ox_] − [*Bs*Fd_red_]) and ([*Bs*FNR_hq_] + 2[*Bs*Fd_ox_] − 2[*Bs*Fd_red_]) were calculated as references without consideration of the absorption changes induced by the complex formations (Figure S7b). Calculated spectra supposed that the spectral changes at around 410 nm and 550–650 nm region give us information about the transitions of the redox state of *Bs*Fd and *Bs*FNR, respectively. The time dependencies of the absorbances at 410 or 413 nm, and 600 nm are presented in Figure 3b,d,f.

**Figure 4:**
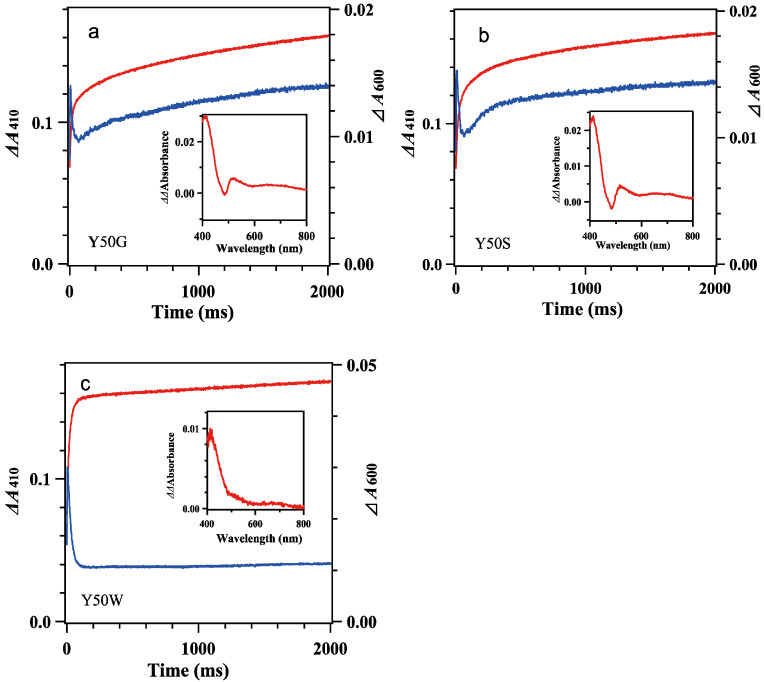
Time dependencies of the *Δ*absorbance at 410 nm (red lines) and 600 nm (blue lines) of mixing oxidized 7.8 μM Y50G (**a**), 8.4 μM Y50S (**b**) and 8.9 μM Y50W (**c**) *Bs*FNR mutants with reduced 74 μM *Bs*Fd in 20 mM HEPES-NaOH buffer (pH 7.0) containing 0.16 μg/mL sodium dithionite at 10 °C. The insets show the difference spectra subtracting the spectrum at 300 ms from that at 2000 ms. Experimental conditions were same as those in Figure 3. The data are an average of five replicates.

Mixing oxidized Y50W mutant (8.9 μM) with excess *Bs*Fd_red_ (74 μM) provided spectral changes (Figure 3e,f). In the spectrum at 1 ms a shoulder at around 480 nm was observable while the absorbance at around 600 nm increased significantly, indicating Y50W *Bs*FNR was reduced to semiquinone form in part within the dead time. With time the absorbance at around 480 nm decreased rapidly (Figure 3e). The absorbance at 600 nm increased and reached a maximum at 5 ms (Figure 3f). Both absorption changes largely responded to the first one-electron reduction reaction, transition from *Bs*FNR_ox_ to *Bs*FNR_sq_ (Figure S7b). The absorbance at 600 nm then turned to decrease because the further reduction of *Bs*FNR_sq_ to *Bs*FNR_hq_ proceeded by the excess *Bs*Fd_red_. The absorbance at 413 nm increased gradually after mixing mainly due to the oxidation of *Bs*Fd_red_ (Figure 3f and Figure S7b). The absorption changes became almost stable at around 200 ms (Figure 3e and Figure 4c). Global analysis of the spectral transition from 0 to 100 ms indicated that the transition was approximated with a two-step sequential reaction model ([A]→[B]→[C] (fast/slow), inset of Figure 3e). The signatures of the estimated spectra of components “B” and “C” in the inset of Figure 3e corresponded well to those of the reference spectra after the first- and second-electron transfer reactions in Figure S7b, respectively. Estimated observed rate constants by the global analysis are indicated in Table 2. The *k*_I_ value is close to the limit of the time resolution of the stopped-flow equipment, thus contains a large error. In the long-term observation, a slow increase was detected at 410 nm (Figure 4c). The difference spectrum in the inset of Figure 4c agreed to the calculated difference spectrum of ([*Bs*Fd_ox_] minus [*Bs*Fd_red_]) (Figure S7a), indicating a slight amount of *Bs*Fd_red_ was oxidized slowly.

The spectral transition of the reaction mixing oxidized 7.8 μM Y50G and 8.4 μM Y50S mutants with excess *Bs*Fd_red_ (74 μM) occurred multiphasically (Figure 3a–d and Figure 4a,b). In the spectra at 1 ms (Figure 3a,c), presences of a shoulder at around 480 nm and a slight increase of the absorbance at around 600 nm indicated the abundance of *Bs*FNR_ox_ within the dead time. With time the absorbance at 600 nm increased and reached a maximum at around 7 ms, then turned to decrease (Figure 3a–d). In contrast to the spectral transition of Y50W (Figure 3f), *ΔΔ*absorbance values of Y50G and Y50S mutants at 410 nm and 600 nm fell into around half of those of Y50W (Figure 3b,d,f). The long-term observations (Figure 4a,b) revealed a presence of slow absorption changes corresponding to the reduction of *Bs*FNR_ox_ to *Bs*FNR_hq_ (inset of Figure 4a,b) [37].

## 4. Discussion

In the previous steady state works of diaphorase assay with NADPH and ferricyanide as electron donor and acceptor, respectively, Y50G and Y50S mutants exhibit a drastic decrease in turnover rate (42.8 s^−1^ and 16.2 s^−1^, respectively [29]), while Y50W mutant provided a rather larger rate constant (183 s^−1^ [29]); yet, these values are less than a quarter of that of WT (1012 s^−1^, [29]). The results in Figure 2 revealed that in the reaction of the three Tyr50 mutants with NADPH the hydride transfer was the rate-limiting step and the rates of the mutants were drastically decreased compared to that of WT [37]. Although the observed hydride transfer rate of phase I was similar among the three Tyr50 mutants, just the half of Y50G and Y50S mutants remained in oxidized state after phase I, and the reduction of the rest proceeded very slowly (phase II) (Figure 2 and Table 1). This delay of the FNR reduction decreases the gross rate of the reaction utilizing reduced FNR to around half: ~50 s^−1^/e and ~20 s^−1^/e for Y50G and Y50S, respectively. These rate constant values are in the same range of those for the diaphorase assay under the saturated amounts of ferricyanide and NADPH concentrations conditions [29]. Because the residues at the NADPH binding site in the other domain and C-terminal extension region (Figure 1) are conserved among the Tyr50 mutants, and the observed hydride transfer rate of WT obtained by the stopped-flow measurement is much faster (~500 s^−1^, [37]), the hydride transfer will limit the turnover rate in the diaphorase assay with Y50G and Y50S mutants.

The biphasic behaviors of Y50G and Y50S mutants were also observed in the *Bs*FNR reduction reaction with reduced *Bs*Fd (Figure 3 and Figure 4). In contrast to the reaction with NADPH, FNR reduction by reduced *Bs*Fd provides similar observed rate constants for both the first and second electron transfer steps in all the three mutants (Table 2). These rate constants are comparable to those of WT ([37], Table 2). Because FNR reduction by Fd proceeds as a sequential one-electron transfer process while FNR reduction by NADPH does as a hydride transfer reaction, the distances between the isoalloxazine ring portion, and the iron-sulfur cluster and nicotinamide ring portion in the complexes may not be modulated drastically by the mutations before reduction.

In the previous report on *Rp*FNR, the FNR reduction with NADPH provide a biphasic kinetic behavior, which is interpreted due to the replacement of NADP^+^ with excess NADPH because an increase in NADPH concentration increases the amount of the reduced FNR and decrease the observed rate constants of the slower phase [16]. In the case of the Tyr50 mutants, however, the rate and *Δ*absorbance of the phase II (*k*_II_ and *A*_II_) were almost independent from NADPH concentration (Table 1), thus phase II is not responsible for the equilibrium [16,37]. Considering that the *Δ*absorbances of phase I and phase II are comparable (Figure 2, Figure 3 and Figure 4, Table 1), most probable catalytic model is that when one protomer is reduced, the conformation of the other protomer is modulated leading a decrease of the reduction rate of the other protomer. In proteins transmission of the conformational changes can complete within microsec. time scale [43], which is rapid enough to fulfill the delay of the reaction of the mutated *Bs*FNRs (~50 s^−1^). In the semiquinone state, the absorption band with a maximum at around 650 nm was omitted in the spectra of the Y50G and Y50S mutants (Figure 3, Figures S1 and S2). The spectra of the fully reduced Tyr50 mutants provide no apparent peak in 400–500 nm region in contrast to the wild type, likely due to the formation of an anionic hydroquinone (Figure 2) [42]. These indicate that the replacement of Tyr50 induced significant changes in the environment of the isoalloxazine ring portion of the reduced states. Yet the structural details in the reduced form are unclear based on the experimental results, molecular dynamics simulation of the reduced WT *Bs*FNR, *Ct*FNR and *Rp*FNR supposes that the reduced FAD induces drastic conformational changes, especially in the C-terminal extension region [42].

**Figure 5:**
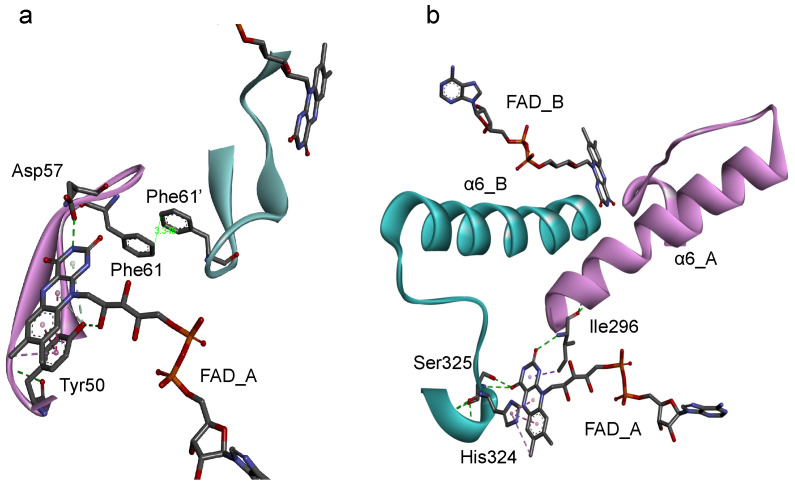
Diagrams representing the symmetric configuration of the *si*-face loop (Tyr50-Arg65) (**a**), and the C-terminal helix 6 and C-terminal extension region (Ile296-Phe329) (**b**) in *Bs*FNR homodimer [PDB ID: 3LZX]. Protomer A and B are colored with pink and blue, respectively.

Based on the protein architecture of *Bs*FNR [14], the isoalloxazine ring portions of FADs in both protomer can affect their conformations each other via (1) the loop region on the *si*-face (Figure 5a) and (2) the helix 6 and following C-terminal extension (Figure 5b). (1) The *si*-face of the isoalloxazine ring is covered with a loop consisting of Tyr50 to Arg65 residues (Figure 5a). This *si*-face loop faces each other, and the Phe61 residues in the loops make a π-π interaction in the homodimer. In this *si*-face loop the key residues interacting with FAD are located: Tyr50 which hydrogen-bonds with the 2′-OH of the ribityl moiety and make a π-π interaction with the isoalloxazine ring portion, and Asp57 which hydrogen-bonds with the N(3)H of the isoalloxazine ring moiety (Figure 5a). In the flavodoxins the *si*-face Tyr contributes to destabilize the FMN hydroquinone state, leading to a decrease in the redox potential of FMN_sq_/FMN_hq_ couple [28,44,45]. In the *Bs*FNR mutants, the omission of Tyr50 in the *si*-face loops may modulate the conformations of the loops and/or FADs in the reduced form. (2) The *re*-face and the pyrimidine ring moiety of the isoalloxazine ring system interact noncovalently with the residues His324 and Ser325 in the C-terminal extension, and Ile296 at the N-terminus of the helix 6 (Figure 5b). Because the two FAD molecules of both protomers can contact via the Ile296—helix 6—C-terminal extension cascades (Figure 5b), conformational changes induced by the changes in redox states of FAD in one protomer can modulate the interaction between FAD, and Ile296 and C-terminal extension of the other protomer. The C-terminal extension of *Bs*FNR is involved in the interactions with both substrates NADP^+^/H and Fd [38]. 

In this work, the pre-steady state reaction analyses of *si*-face Tyr50 mutants of *Bs*FNR revealed that the replacements with Trp, Gly and Ser decrease the hydride transfer rate in the reaction with NADPH, and replacement with Gly and Ser induced abnormal biphasic kinetic behavior in the FNR reduction reactions. Considering that *Bs*FNR forms a homodimer and the *Δ*absorbances of both kinetic phases are comparable, a reduction of one protomer may induces conformational changes of the other protomer which decrease the rate for the reduction of the other protomer in these mutants. In homodimeric *Bs*FNR, the presence of the *si*-face Tyr stabilizes the positioning of the isoalloxazine ring portion and/or a conformation of the polypeptide in the reduced state, which increase the turnover rate of the reaction with physiological substrates.

## Figures and Tables

**Table 1:** Kinetic constants of the reaction mixing oxidized Tyr50 mutants with NADPH and *S*-NADPD.

[NADPH]		100 μM ^a^	200 μM ^a^	300 μM	500 μM ^a^	100 μM *S*-NADPD ^b^
Y50G	*k*_I_	46.6 ± 0.3 (43.7 ± 1.5)	49.9 ± 0.3 (48 ± 2)	49.9 ± 0.4 (49 ± 2)	50.7 ± 0.4 (50 ± 4)	9.48 ± 0.03 (9.8 ± 0.4)
	*A*_I_	0.0302 ± 0.0001	0.0290 ± 0.0001	0.0289 ± 0.0001	0.0300 ± 0.0001	0.0262 ± <0.0001
	*k*_II_	2.01 ± 0.02 (1.88 ± 0.07)	1.96 ± 0.01 (1.89 ± 0.09)	1.97 ± 0.02 (1.92 ± 0.06)	1.81 ± 0.02 (1.76 ± 0.05)	0.861 ± 0.004 (0.88 ± 0.04)
	*A*_II_	0.0221 ± <0.0001	0.0249 ± <0.0001	0.0251 ± <0.0001	0.0267 ± <0.0001	0.0318 ± <0.0001
Y50S	*k*_I_	19.39 ± 0.09 (16.5 ± 0.4)	19.24 ± 0.08 (16.2 ± 0.3)	19.59 ± 0.08 (15.7 ± 0.5)	19.25 ± 0.08 (16.0 ± 0.4)	-
	*A*_I_	0.0411 ± 0.0001	0.0422 ± 0.0001	0.0408 ± <0.0001	0.0429 ± <0.0001	-
	*k*_II_	2.17 ± 0.02 (1.21 ± 0.03)	2.14 ± 0.02 (1.12 ± 0.04)	2.29 ± 0.02 (1.10 ± 0.05)	2.19 ± 0.02 (1.04 ± 0.04)	-
	*A*_II_	0.0224 ± <0.0001	0.0249 ± <0.0001	0.0260 ± <0.0001	0.0255 ± <0.0001	-
Y50W	*k*_I_	37.4 ± 0.13 (35.9 ± 0.6)	37.5 ± 0.12 (36.2 ± 1.1)	37.4 ± 0.12 (33.5 ± 0.7)	38.4 ± 0.14 (36.8 ± 1.4)	3.757 ± 0.005 (3.69 ± 0.04)
	*A*_I_	0.0622 ± 0.0001	0.0618 ± 0.0001	0.0611 ± 0.0001	0.0613 ± 0.0001	0.0651 ± <0.0001

Observed rate constant (*k*) and amplitude (*A*) of each phase (I or II) obtained by fitting with a single- or double-exponential decay function against absorption changes at 460 nm are presented with ± one standard deviation values. The residuals of the fitting are presented in Figure S4. Observed rate constants with a global analysis using one or two-step sequential reaction model ([A]→[B]) or ([A]→[B]→[C] (fast/slow))) are indicated in the parentheses with ± one standard deviation values. ^a^: original absorption data are indicated in Figure 2. ^b^: original absorption data are indicated in Figure S3.

**Table 2:** Observed rates for the *Bs*FNR reduction by a reduced *Bs*Fd.

	Y50G ^1^	Y50S ^1^	Y50W ^1^	WT ^2^
*k*_I_	440 ± 80	483 ± 6	315 ± 4	>500
*k*_II_	51 ± 3	44.1 ± 0.7	47.1 ± 0.9	66 (80 μM Fd)

^1^ Spectroscopic data utilized for the analyses are represented in Figure 3. ^2^ The data are cited from [37].

## Data Availability

The author confirms that the data supporting the findings of this study are available within the article and Supplementary Materials. Raw data are available from the corresponding author upon reasonable request.

## Supplementary Materials

The following are available online at https://www.mdpi.com/article/10.3390/antiox12091741/s1, Figure S1: Transient absorption spectra of oxidized Y50 mutants in the absence of NADPH, Figure S2: Photoreduction of Y50G *Bs*FNR mutant in the presence of 5 mM EDTA, Figure S3: Transient absorption spectra of mixing Y50G and Y50W *Bs*FNR mutants with 100 μM *S*-NADPD, Figure S4: Residuals of fitting the absorbance change at 460 nm in mixing NADPH with oxidized Y50 mutants, Figure S5: Transient absorption spectra of the reaction mixing reduced *Bs*FNR mutants with NADP^+^, Figure S6: Transient spectra induced by mixing reduced *Bs*Fd with Y50 mutants, Figure S7: Calculated spectra of Y50G mutant in three different redox states and elicited by coupled redox reactions between Y50G *Bs*FNR_ox_ and *Bs*Fd_red_.
