# Supplementary material for: The Role of the si-Face Tyrosine of a Homodimeric Ferredoxin-NADP+ Oxidoreductase from Bacillus subtilis during Complex Formation and Redox Equivalent Transfer with NADP+/H and Ferredoxin
Source: Antioxidants (Basel). 2023 Sep 8;12(9):1741. doi: 10.3390/antiox12091741 (PMC10526003; doi:10.3390/antiox12091741)
Supplement: Supplementary file 1 [file antioxidants-12-01741-s001.zip › antioxidants-2538813-supplementary.pdf]

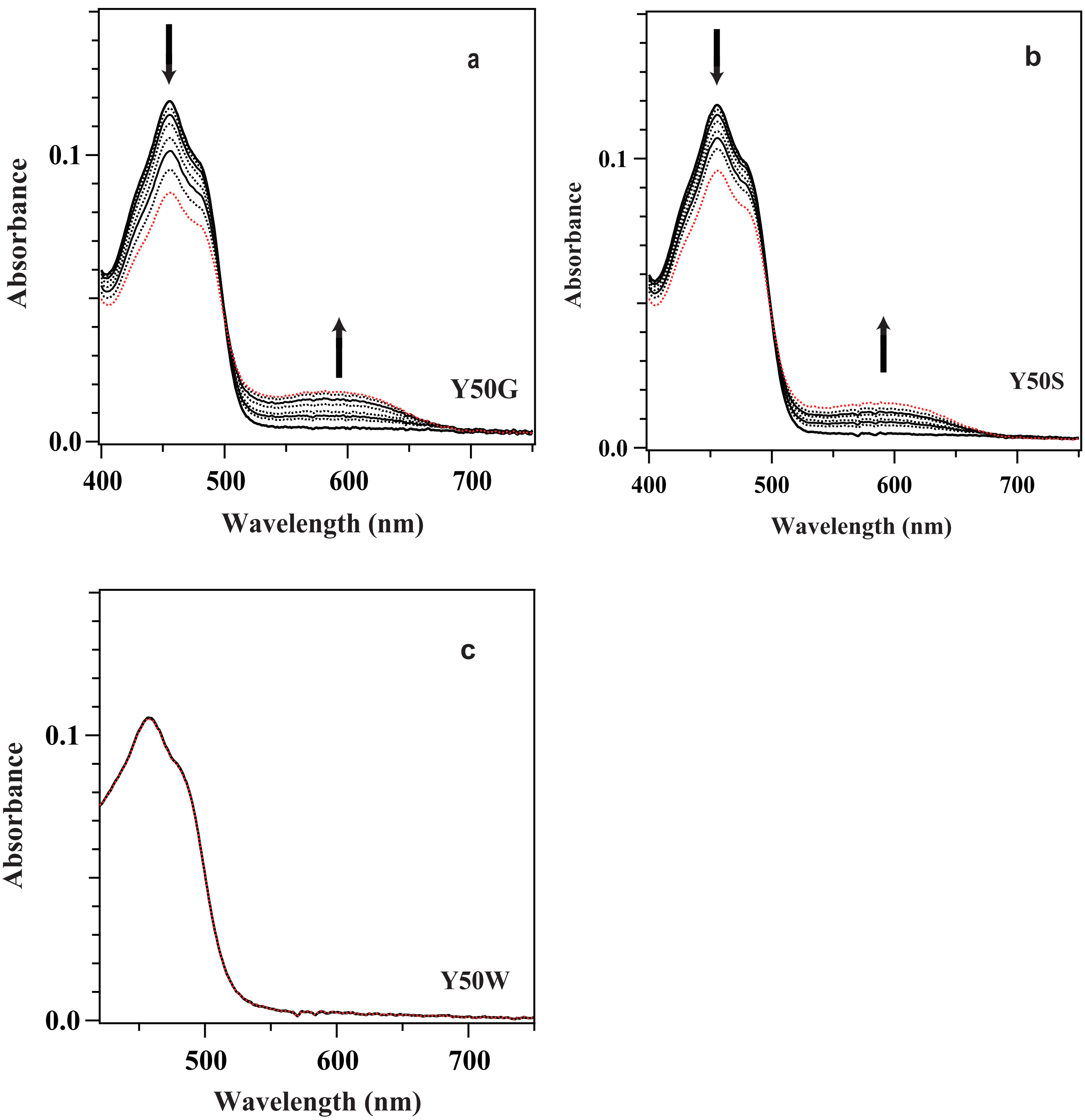

Figure S1 Transient absorption spectra for mixing oxidized 9.1 μM Y50G (**a**), 9.1 μM Y50S (**b**) and 8.9 μM Y50W (**c**) *Bs*FNR mutants with 20 mM HEPES-NaOH buffer (pH 7.0) at 10°C. In (**a**) and (**b**), continuous lines indicate the spectra at 1, 100 and 1000 ms and thin dotted lines at 50, 200, 500, 2000 and 3997 ms from top to bottom at 450 nm. The spectra at 3997 ms are indicated in red. In (**c**), the spactra at 1 and 100 ms are indicated with continuous lines. The spectra at 2000 ms is indicated with red dotted line. The data are an average of five replicates.

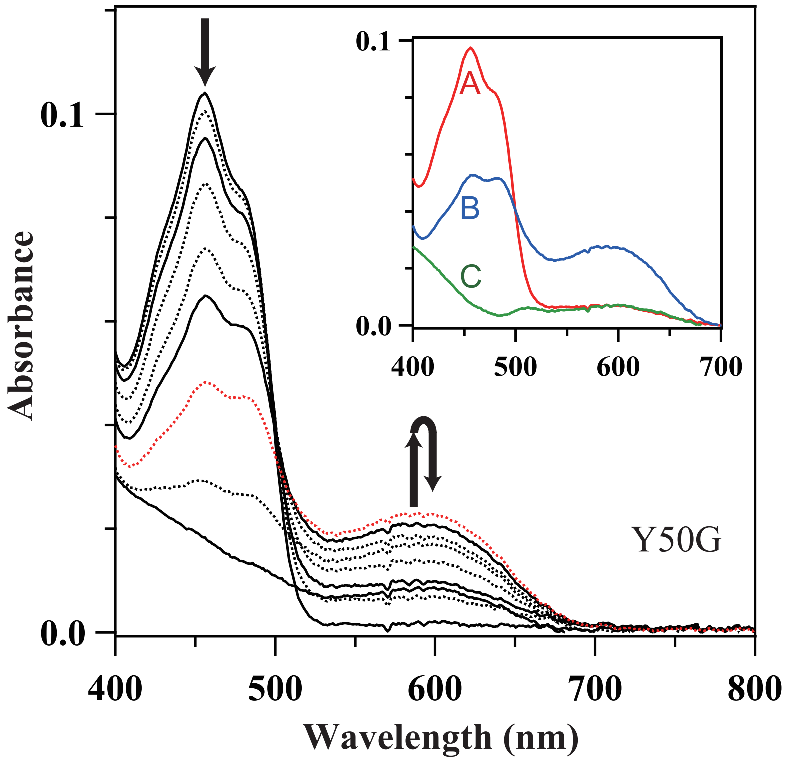

Figure S2 Photoreduction of Y50G *Bs*FNR mutant in the presence of 5 mM EDTA in 20 mM HEPES-NaOH buffer (pH 7.0) at 10°C. Thin continuous lines represent the spectra at 0 s, 100 ms, 1 s and 10 s, and thin dotted lines at 50 ms, 200 ms, 500 ms, 2 s and 5 s from top to bottom at 450 nm. The spectrum at 2 s is in red coloration. The inset shows the spectra of kinetic components estimated by a global analysis of the transient spectra with a two-step sequential reaction model ([A]→[B]→[C] (fast/slow)). The data are an average of five replicates.

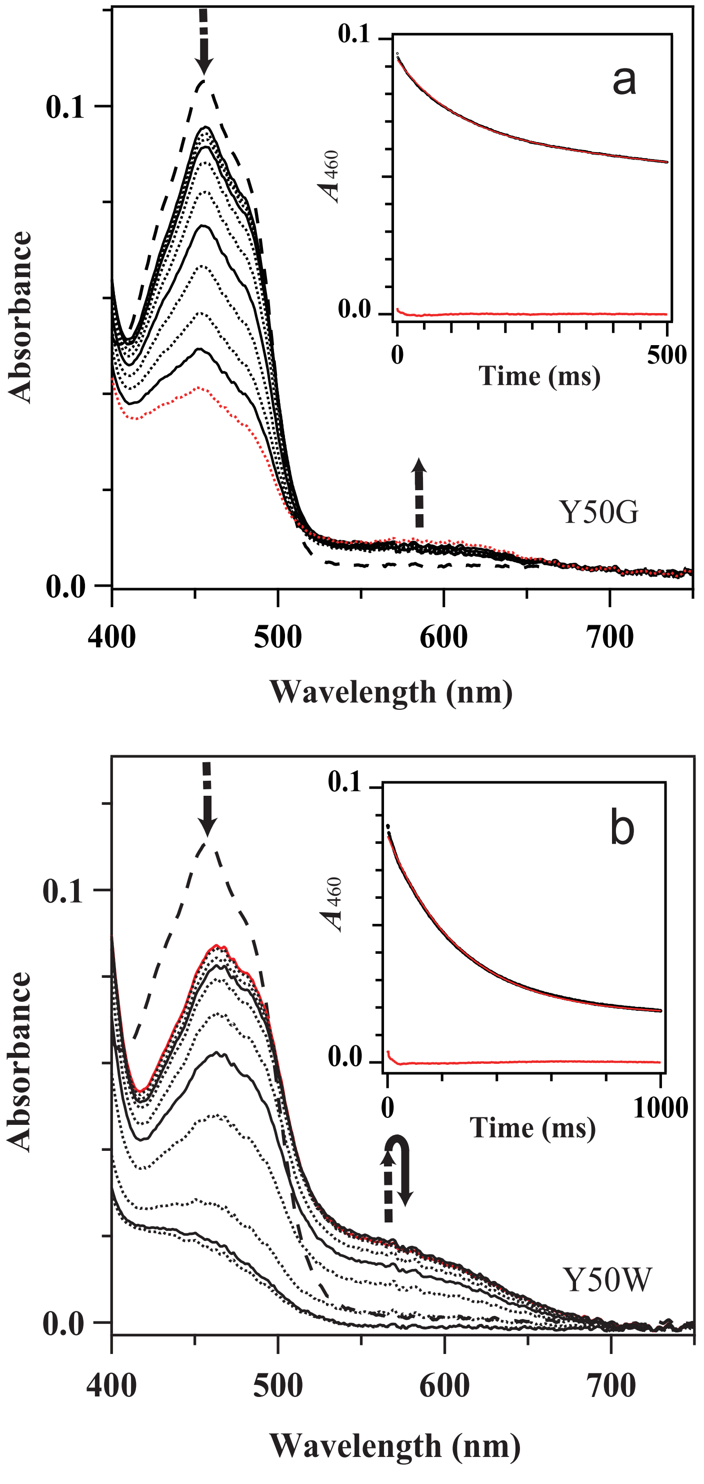

Figure S3 Transient absorption spectra of the reaction of mixing oxidized 8.0 µM Y50G (**a**), and 9.4 µM Y50W (**b**) *Bs*FNR mutants with 100 µM *S*-NADPD in 20 mM HEPES-NaOH buffer (pH 7.0) at 10°C. Thick broken lines indicate the spectra of the oxidized mutants. Thin continuous lines represent the spectra at 1, 10, 100, and 1000 ms and thin dotted lines at 2, 5, 20, 50, 200, 500 and 2000 ms from top to bottom at 450 nm. The spectra at 2000 ms in (**a**) and at 1 ms in (**b**) are indicated with red coloration. Insets: time course of the absorption changes at 460 nm. The fitted curves with a one or two-component exponential function are indicated as red lines. The residuals are indicated as red lines at the bottom. The data are an average of five replicates.

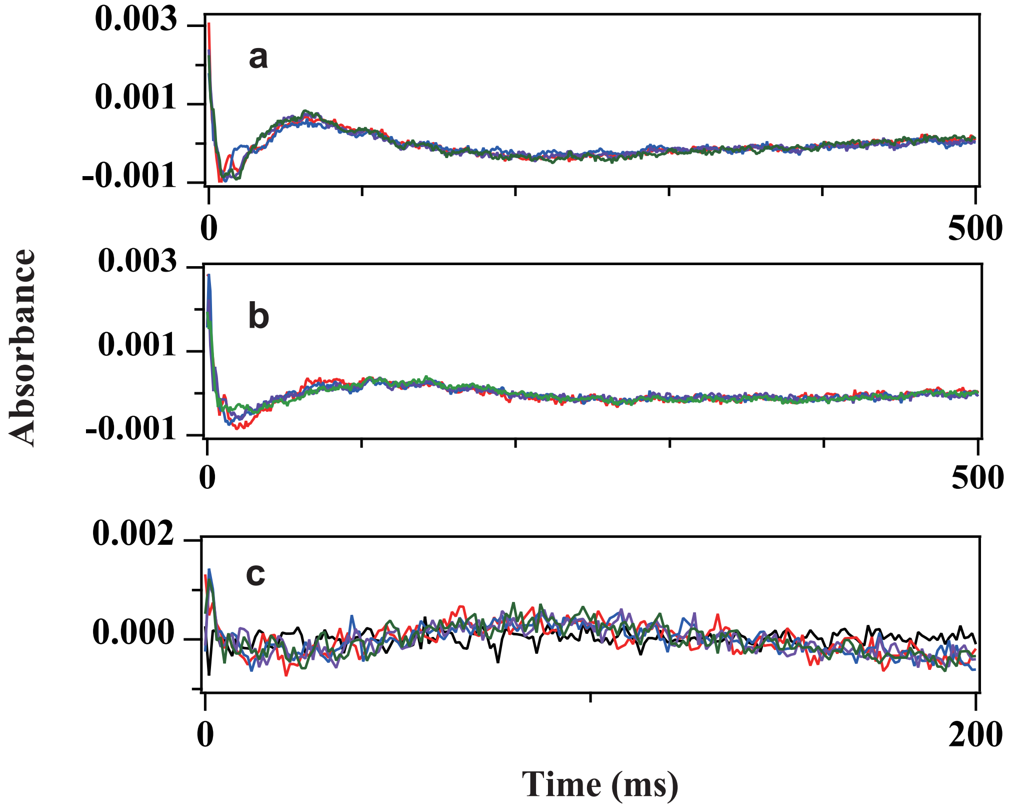

Figure S4 The residuals of fitting the absorption changes at 460 nm in mixing NADPH with oxidized Y50G (**a**), Y50S (**b**) and Y50W (**c**) *Bs*FNRs with 0 (black), 100 (red), 200 (blue), 300 (purple) and 500 μM (green) NADPH using a single or double exponential decay function. The original spectroscopic data are represened in Figure 2. Obtained kinetic parameters are represented in Table 1.

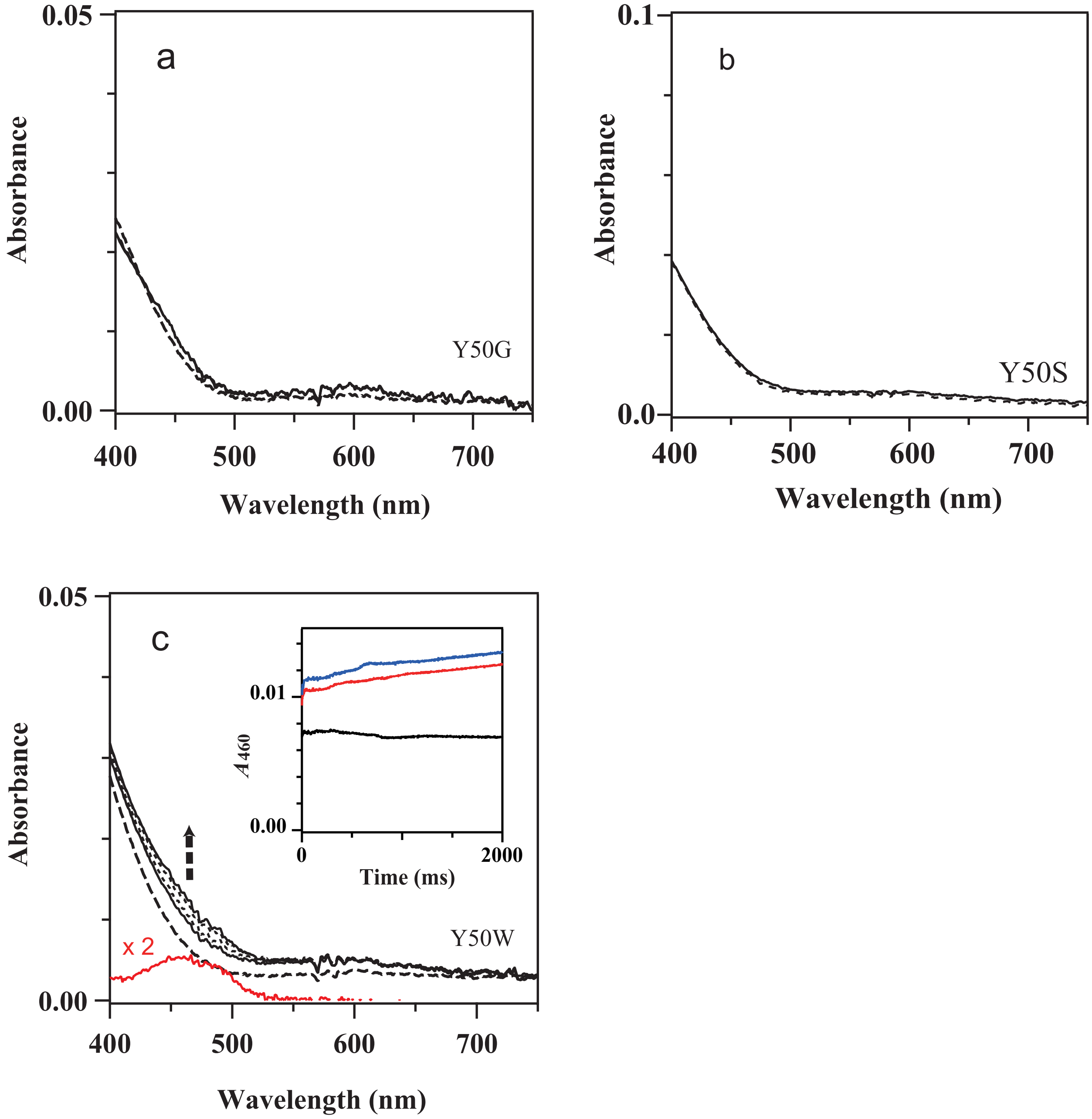

Figure S5 (**a-c**) Absorption spectra induced by mixing reduced 7.1 µM Y50G (**a**), 9.1 µM Y50S (**b**) and 6.9 µM Y50W (**c**) *Bs*FNR mutants with 500 µM $NADP^+$ in 20 mM HEPES-NaOH buffer (pH 7.0) at 10°C. The spectra of the reduced mutants are indicated with thick broken lines. The spectra at 1 ms and 1000 ms of Y50G (**a**), 1000 ms of Y50S (**b**), and 1 ms and 2000 ms of Y50W (**c**) mutants after mixing are shown with thin continuous lines, and the spectra at 100 and 1000 ms of Y50W mutant (**c**) are indicated with broken lines from the bottom to the top at 450 nm. In (**c**) a difference spectrum subtracting the spectrum at 1 ms from at 2000 ms is indicated with red continuous line and two-fold magnification. The inset in (**c**) shows the time dependency of the absorbance at 460 nm of mixing reduced Y50W mutant with 0 (black), 100 (red) and 500 µM (blue) $NADP^+$. The data are an average of five replicates.

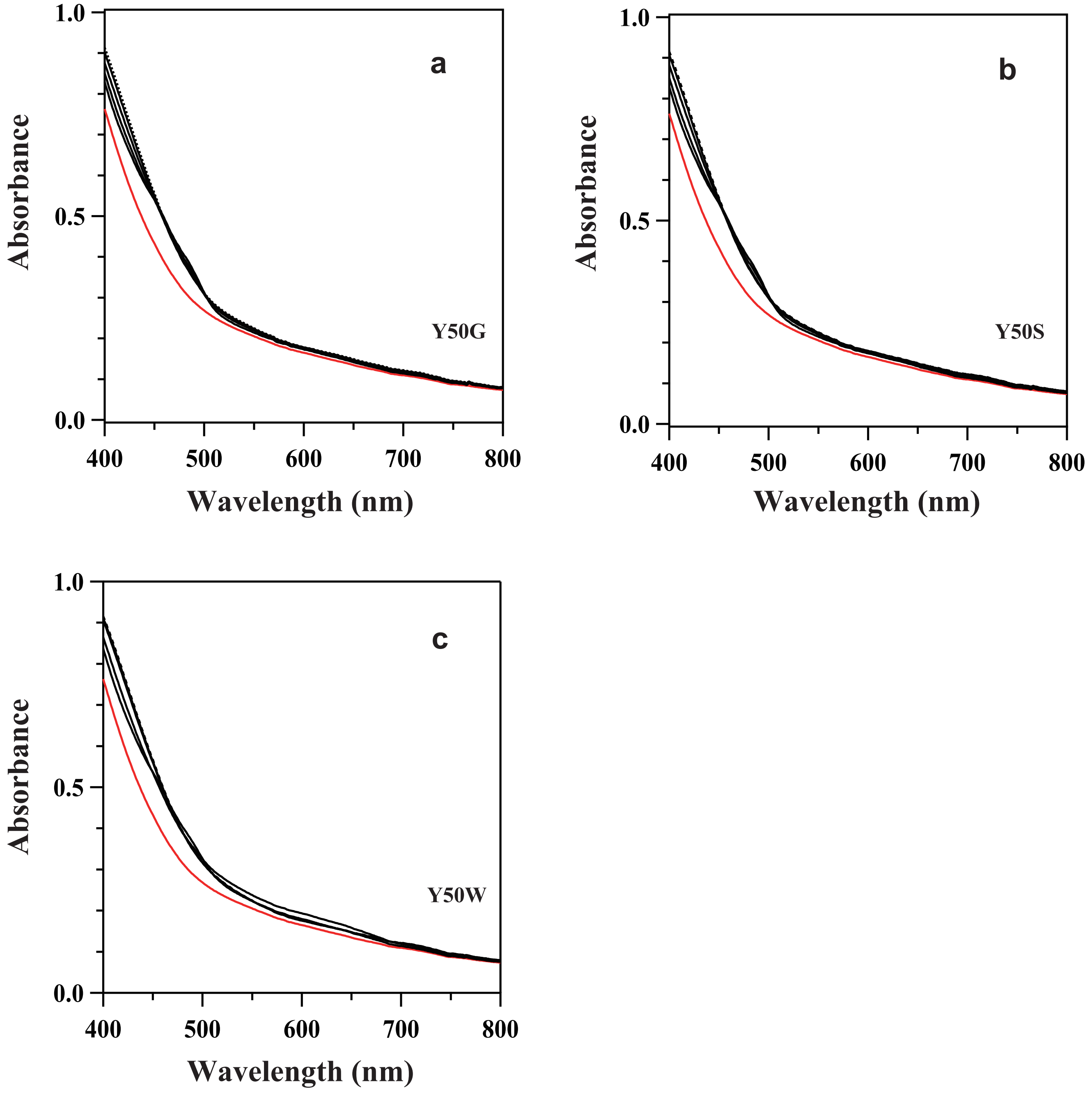

Figure S6 Transient spectra induced by mixing reduced *Bs*Fd (74 µM) with oxidized 7.8 µM Y50G (**a**), 8.4 µM Y50S (**b**) and 8.9 µM Y50W (**c**) *Bs*FNR mutants in 20 mM HEPES-NaOH buffer (pH 7.0) containing 0.16 µg/ml sodium dithionite at 10˚C under anaerobic conditions. The spectra at 1, 10, 100 and 1000 ms are indicated with continuous lines, and at 2000 ms with broken lines. Spectrum of the reduced *Bs*Fd (74 µM) obtained by mixing with buffer is indicated with red lines.

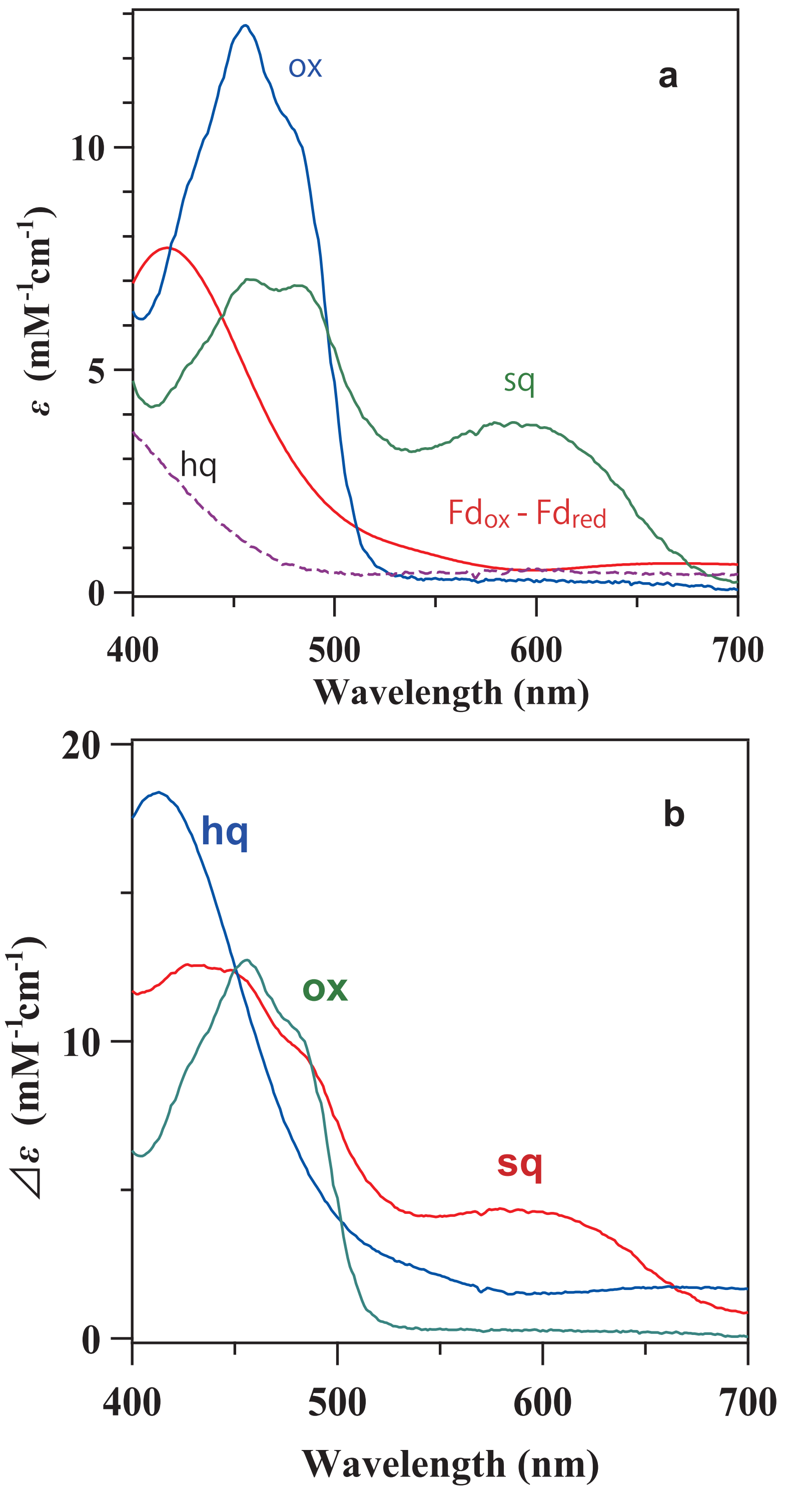

Figure S7(a) Spectra of oxidized (blue), semiquinone (green) and fully reduced (purple) forms of Y50G *Bs*FNR mutant and a difference spectrum of ([*Bs*Fd$_{ox}$] minus [*Bs*Fd$_{red}$]) (red) under anaerobic conditions. The spectra of Y50G were estimated using the data represented in Figures 2a, S5a and S2. The difference spectrum of *Bs*Fd was obtained from [37].

(**b**) Calcurated spectra elicited by coupled redox reactions between Y50G *Bs*FNR$_{ox}$ and *Bs*Fd$_{red}$. The spectra corresponding to the reduction of FNR$_{ox}$ to FNR$_{sq}$ ([*Bs*FNR$_{sq}$] + [*Bs*Fd$_{ox}$] - [*Bs*Fd$_{red}$]), the reduction of FNR$_{sq}$ to FNR$_{hq}$ ([*Bs*FNR$_{hq}$] + 2×[*Bs*Fd$_{ox}$] - 2×[*Bs*Fd$_{red}$]) as well as [*Bs*FNR$_{ox}$] are indicated with red, blue and green continuous lines, respectively.
